# Automatic tracking of cells for video microscopy in patch clamp experiments

**DOI:** 10.1186/1475-925X-13-78

**Published:** 2014-06-20

**Authors:** Helton M Peixoto, Hermany Munguba, Rossana MS Cruz, Ana MG Guerreiro, Richardson N Leao

**Affiliations:** 1 Brain Institute, Federal University of Rio Grande do Norte, 2155, 59056-450 Natal - RN, Brazil; 2 Federal Institute of Education, Science and Technology of Paraiba, Joao Pessoa - PB, Brazil; 3 Department of Computer Engineering and Automation, Federal University of Rio Grande do Norte, Natal - RN, Brazil; 4 Department of Neuroscience, Uppsala University, Uppsala, Sweden

**Keywords:** Neurons, Fluorescent proteins, Photodamage, Patch-clamp, Image tracking, Mask overlay

## Abstract

**Background:**

Visualisation of neurons labeled with fluorescent proteins or compounds generally require exposure to intense light for a relatively long period of time, often leading to bleaching of the fluorescent probe and photodamage of the tissue. Here we created a technique to drastically shorten light exposure and improve the targeting of fluorescent labeled cells that is specially useful for patch-clamp recordings. We applied image tracking and mask overlay to reduce the time of fluorescence exposure and minimise mistakes when identifying neurons.

**Methods:**

Neurons are first identified according to visual criteria (e.g. fluorescence protein expression, shape, viability etc.) and a transmission microscopy image Differential Interference Contrast (DIC) or Dodt contrast containing the cell used as a reference for the tracking algorithm. A fluorescence image can also be acquired later to be used as a mask (that can be overlaid on the target during live transmission video). As patch-clamp experiments require translating the microscope stage, we used pattern matching to track reference neurons in order to move the fluorescence mask to match the new position of the objective in relation to the sample. For the image processing we used the Open Source Computer Vision (OpenCV) library, including the Speeded-Up Robust Features (SURF) for tracking cells. The dataset of images (n = 720) was analyzed under normal conditions of acquisition and with influence of noise (defocusing and brightness).

**Results:**

We validated the method in dissociated neuronal cultures and fresh brain slices expressing Enhanced Yellow Fluorescent Protein (eYFP) or Tandem Dimer Tomato (tdTomato) proteins, which considerably decreased the exposure to fluorescence excitation, thereby minimising photodamage. We also show that the neuron tracking can be used in differential interference contrast or Dodt contrast microscopy.

**Conclusion:**

The techniques of digital image processing used in this work are an important addition to the set of microscopy tools used in modern electrophysiology, specially in experiments with neuron cultures and brain slices.

## Introduction

Patch-clamp experiments in cultures or brain slices require a careful assessment of targeted cells before placing the patch electrode [1]. Consequently, the microscope stage is constantly moved in both xy axis as in z (focus) and targeted cells can often be missed during the patch electrode lowering. In addition, with the discovery and cloning of the green fluorescent protein (GFP) [2], fluorescence optical imaging permits not only the identification of genetically distinct neuronal populations but also queries for intracellular changes of ion concentrations or membrane potential in subcellular compartments that are unreachable to electrodes [3-5]. The rapid development and widespread usage of fluorescent probes demand the creation and optimisation of fluorescent microscopy techniques. To minimise bleaching and photodamage, modern microscope objectives have a very high numerical aperture and more sensitive detection systems that virtually approach the ideal photon detection limit by converting every photon reaching the sensor into an electron. For example, some back-illuminated cooled EM-CCD (Electron Multiplying Charge Coupled Device) cameras can have quantum efficiencies (the ratio between electron-hole pairs produced by photons colliding with the sensor and the total number of photons that actually collided with the sensor) very close to 100%. In addition to the optimisation of objectives and detectors, modern emission and excitation filters achieve sharp and efficient cut off of unwanted wavelengths, while passing almost with no loss the desired light spectrum. Yet, detection of fluorescence emanating from synthetic compounds or proteins still requires high intensity excitation, which damages the tissue (when applied for long periods) especially in epifluorescence systems, often used in patch-clamp experiments [6,7].

Typically, visualisation of (unstained) neurons in in vitro patch-clamp experiments is best achieved with Differential Interference Contrast (DIC) [8]. DIC uses a set of filters (analysers) and polarisers between the condenser and the sample, as well as between the objective and the detection system [8]. Hence, the presence of a filter and an analyser between the objective and the camera severely impairs epifluorescence imaging; besides, these components have to be mechanically removed from the light path when switching between DIC and fluorescence modes. Often, the removal of these optical components can cause vibrations that can damage the patch pipette contact with the neuron. Alternatively, an illumination technique known as gradient contrast (Dodt contrast) requires no optical components between the detector and the objective, but does not reach the level of detail obtained with DIC [9]. In both methods, cells labeled with fluorescent probes are localised by the prolonged and repetitive exposure to the excitation light. Due to low levels of expression and/or efficiency of fluorescent probes, intense excitation (that often damages the tissue) is required for cell visualisation [6]. The advent of optogenetics has also brought to light another pitfall of prolonged light exposure for fluorescence imaging [10]. Optogenetic effectors (e.g. channelrhodopsin) are often linked to fluorescent proteins to allow the identification of neurons expressing these rhodopsins [7,10]. The wavelengths used in the excitation of fluorescent proteins associated to the rhodopsin also provoke the activation of the optogenetic proteins causing (unwanted) neuron activation [7]. Hence, patch-clamp experiments involving fluorescent imaging and/or optogenetic neuronal activation can benefit dramatically from the minimization of exposure to high intensity light sources. In this work, we use digital image processing techniques like Speeded-Up Robust Features (SURF) [11] and image overlay. Computational development was also performed using C++ programming language and Open Source Computer Vision (OpenCV) library. This allowed a considerable reduction of the exposure time of the tissue to excitation light and an easier manipulation of target cells, providing improvements in experiments with fluorescent guided patch-clamp.

## Materials and methods

Experiments using C57B6, or *Gad*2^*t* *m*2(*c* *r* *e*)*Z* *j* *h*/*J*^ (Jackson Labs) crossed with *Gt*(*ROSA*)26*Sor*^*t* *m*14(*C* *A* *G*−*t* *d* *T* *o* *m* *a* *t* *o*)*H* *z* *e*^ (Allen Brain Institute) (*Gad*2^*t* *d* *T* *o* *m*^) were conducted in accordance with the Uppsala University and Federal University of Rio Grande do Norte guidelines for care and use of laboratory animals. All efforts were made to minimise the suffering and the number of animals used.

### Cell culture

Primary cultures of hippocampal neurons for transfection with the genetic encoded calcium indicator yellow cameleon (gift from Takeharu Nagai, Sapporo University) [12] were prepared from mouse pups 0–2 days old. Hippocampi were dissected in ice-cold PBS 10 *mM* glucose and transferred to a similar solution containing 0.5 *mg*/*ml* papain and 10 *ul*/*ml* DNase for 30 min at 37°C. The hippocampi were triturated with glass pipettes and the resulting cell suspension was seeded in plates with coverslips precoated with both poly-L-lysine and laminin.

Cultures were plated in Neurobasal-A Medium, supplemented with 2% B27 (NB/B27), 1 *mM* Na-pyruvate, 2 *mM* L-glutamine, and 100 *U*/*ml* penicillin + 100 *mg*/*ml* streptomycin (1x PEST). Cultures went through calcium phosphate transfection 4–6 days after plating. The cells were allowed to grow until analysis of expression or electrophysiology.

### Hippocampus slices

P21-P28 hippocampal slices were obtained as described in [13]. In summary, brains were rapidly removed and placed in ice-cold sucrose/artificial cerebrospinal fluid (ACSF), in mM: *KCl*, 2.49; *NaH*_2_*PO*_4_, 1.43; *NaHCO*_3_, 26; glucose, 10; sucrose, 252; *CaCl*_2_, 1.0; *MgCl*_2_, 4.0. Horizontal slices containing the hippocampus were obtained using a vibratome and subsequently moved to a submerged holding chamber containing ACSF (in mM: *NaCl*, 124; *KCl*, 3.5; *NaH*_2_*PO*_4_, 0.25; *MgCl*_2_, 1.5; *CaCl*_2_, 1.5; *NaHCO*_3_, 30; glucose, 10), bubbled with 95% *O*_2_/5% *CO*_2_, where they were kept until being moved to the microscope stage.

### Video microscopy

Images analysed in this work are derived from neuron cultures and brain slices and were collected with different levels of brightness, focus and displacement. Sample images were obtained using a standard fixed-stage DIC and Dodt contrast based microscopes (BX51WI, Olympus) and a 40x (0.8NA) water immersion objective (Olympus). Yellow or red fluorescence images were obtained either using a 200*W* metal halide lamp (Prior, UK) or a custom mounted 440 *nm* LED array [5] using, respectively, ET436/20x and ET545/30x excitation filters (Chroma, USA), T455LP and T570LP dichroics (Chroma) and 542/27-25 (Semrock, USA) and ET620/60m (Chroma, USA) emission Filters. A single EM-CCD (either a Luca S or an Ixon 897, Andor, Ireland) camera was used for transmission and fluorescence detection. DIC optics used was the Olympus standard and for the patterned illumination required to the Dodt contrast method, we used a commercial Dodt contrast tube between the transmission light source and the condenser (Luigs and Neumann, Germany).

### Image processing techniques

**Figure 1:**
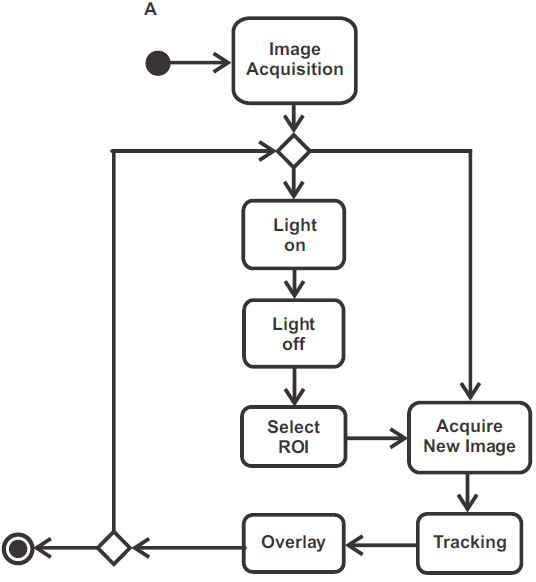
**UML activity diagram.** The first step of the diagram is the acquisition of frames (30 fps) from the camera that is coupled to the microscope. Light on means that the fluorescent light is turned on, and therefore the image displays information of cell fluorescence. Light off means that the fluorescent light is turned off. Select ROI (Region of Interest) permits the user to select a ROI in the image, to be used in neuron tracking and overlay steps. Acquire New Image performs the acquisition of the next frame. Tracking step performs ROI tracking in subsequent images. Overlay means to accomplish blending between the current image and the fluorescence information (acquired in light on step) of ROI during tracking.

The computational algorithms utilized in this work were implemented with C++ programming language and OpenCV for real time computer vision. Figure 1 shows an activity diagram UML (Unified Modeling Language) indicating the steps for image mask overlay and neuron tracking. For mathematical purposes, consider an image as a function of two variables *f*(*x*,*y*) where *x* and *y* are spatial (plane) coordinates and the value at a given point *I*(*x*,*y*) is the amplitude of the pixel.

### Tracking with SURF

SURF method is a scale and rotation-invariant detector and descriptor used in object recognition. This technique that shows high repeatability, distinctiveness and is computationally efficient [11]. The operation of SURF initially consists in selects interest points at different locations within the images (e.g. blobs). Subsequently, it creates a feature vector which includes characteristics of points neighboring the region of interest. Finally, matching is performed by measuring distances between vectors using formulae like the Mahalanobis or Euclidian metric.

#### Interest points

SURF detector is based on the calculation of the determinant of Hessian matrix (H), that allows to find blob-like structures at locations from the maximum value of the determinant. This can be calculated directly according to (1), but it is not computationally recommended. Thus, it can be calculated by the approximation given in (2), where the Hessian matrix is accomplished the function space **x**=(*x*,*y*) and *σ* scale.

(1) $$H(f(x,y))=\left[\begin{array}{cc} \frac{\partial^{2}f}{\partial x^{2}} & \frac{\partial^{2}f}{\mathrm{\partial x\partial y}}\\ \frac{\partial^{2}f}{\mathrm{\partial y\partial x}} & \frac{\partial^{2}f}{\partial y^{2}} \end{array}\right]$$

(2) $$H(\text{x},\sigma )=\left[\begin{array}{cc} L_{\text{xx}}(\text{x},\sigma ) & L_{\text{xy}}(\text{x},\sigma )\\ L_{\text{yx}}(\text{x},\sigma ) & L_{\text{yy}}(\text{x},\sigma ) \end{array}\right]$$

and

(3) $$L_{\text{xx}}(\text{x},\sigma )=I(\text{x})*\frac{\partial^{2}}{\partial x^{2}}g(\sigma )$$

**Figure 2:**
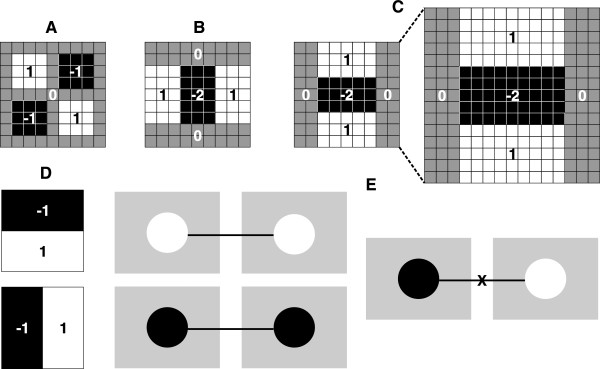
**Filters and types of matching used by SURF.(A)**, **(B)**, and **(C)** Approximation of Gaussian second order partial derivatives using box filters: *D*_*xy*_ in xy-direction, *D*_*xx*_ in x-direction and y-direction in *D*_*yy*_, respectively, with increased kernel size from (9×9) to (15×15) in **(C)**. **(D)** Haar wavelet filters in response to *dx* (top) and *dy* (bottom) directions with (*σ*=2*s*) kernel size. **(E)** Candidate interest points to perform matching are combinations between circular areas with the same contrast and, the oposite are not considered.

Where the *L*_*x* *x*_(**x**,*σ*) in (3) is the convolution of the second order Gaussian derivative $\frac{\partial^{2}}{\partial x^{2}}g(\sigma )$ with the image at point *I*(**x**) and similarly to *L*_*y* *y*_ and *L*_*x* *y*_. The images are represented by performing an integral calculus based on Viola and Jones method [14], and the second derivative of gaussian kernel is approximated by the box filter shown in Figure 2A-C.

Then the determinant of the Hessian matrix can be calculated by (4), and *w* is a weight used to balance the equation. That is, the blob response at location *I*(**x**).

(4) $$\text{Det}(H)\approx D_{\text{xx}}D_{\text{yy}}-{\left(wD_{\text{xy}}\right)}^{2}$$

Surf allows to find interest points in different scales. The idea of scale-space is implemented by changing the size (eg. 9×9, 15×15, 21×21 and 27×27) of the kernel used in the convolutional process. Thus, to locate and map the interest points in both space and scale, it is applied the 3D Non Maximum Suppression (NMS) technique to a (3×3×3) neighborhood as sub-pixel accuracy as can be seen in [15,16].

#### Feature vector and Matching

The creation of a feature vector (*V*) 64-dimensional (5) is based on information from descriptors of a area around the interest point. These descriptors are the result of applying filters (Haar wavalets) Figure 2D centered around (4×4 regular subregions) the interest points of the image, and provide the gradient in *x* and *y* directions. This allows invariance to rotation, translation and brightness during matching [15].

(5) $$V=\left(\sum \text{dx},\sum \text{dy},\sum |\text{dx}|,\sum |\text{dy}|\right)$$

In matching step, it is verified the sign of the Laplacian matrix (6) with no extra computational cost, since this information is previously known. This allows the comparison of two similar types of contrast (ie dark or clear blob-types) Figure 2E.

(6) $$\nabla^{2}L=\text{tr}(H)=L_{\text{xx}}(\text{x},\sigma )+L_{\text{yy}}(\text{x},\sigma )$$

The Laplacian of a Hessian matrix is the sum of the diagonal elements. Finally, the algorithm seeks for the smallest Euclidean distance between the vectors, that is, the pairs most likely to be similar.

### Alpha blending

Two-dimensional domain pixel transforms are performed in one or more input images, producing an output image. This operation is given by:

(7) $$g(x,y)=h(f_{0}(x,y),\ldots ,f_{n}(x,y))$$

where *g*(*x*,*y*) is the result of the transform applied on the input images *f*(*x*,*y*).

Alpha blending technique can perform blending between 2 images of different sizes, for example, a ROI and an image frame. In this way, it is possible to combine the two images and overlay one over another. Alpha blending expression is shown in (8):

(8) $$g(x,y)=\alpha f_{0}(x,y)+(1-\alpha )f_{1}(x,y)$$

where *α* must be a value between 0 and 1 and it represents the blending opacity level. *f*_0_(*x*,*y*) is a ROI and *f*_1_(*x*,*y*) is the image [17].

In this work, after matching step, the geometric transformation (homography matrix) between images (ROI and frame) can be estimated using the RANSAC (RANdom SAmple Consensus) algorithm [18]. In this way, overlay is performed considering the homography matrix limits, which makes the scenario even more natural.

## Results

As shown in Table 1, the dataset of analyzed images consists of eight sets equally divided between neuron cultures and brain slices. Each set is composed by a series of 90 images, totalizing 360 images of each type. The computer used in the experiments is a 64-bit (x64) Intel Core i5 with 8 GB of RAM, OS Ubuntu linux 12.04.

**Table 1:** Image dataset

**Sets 1,2,3,4**	**Number of images**	**Image size**	**ROI size**
Culture	360	640×480	140×155
Slice	360	640×480	155×140

### 

#### Image acquisition environment

**Figure 3:**
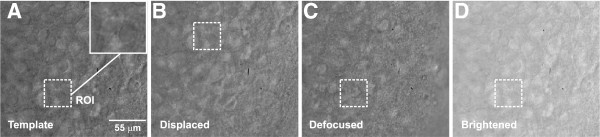
**Images of brain slices in different conditions.(A)** Slice image used as a template for the selection of a ROI. **(B)** ROI tracking during microscope displacements (xy axis). **(C)** ROI tracking during image defocusing. **(D)** ROI tracking under high brightness conditions due gamma increase.

The diversity of the environment where images were obtained requires that image processing techniques are reliable to the point of allowing the user to maintain the position of the ROI in real time during the patch-clamp experiments, which can be difficult just by performing a simple live acquisition. Figure 3 exemplifies the use of SURF for tracking a ROI in adverse environment conditions, such as displacement, defocusing and brightness, during image acquisition.

The open source software was implemented in C++ and OpenCV with no restriction for distribution and there are future plans to include electrophysiology data acquisition routines in the imaging package described here. This software is released under the GNU General Public License and the latest development sources are available online (https://github.com/neurodynamics/neuronTracking).

#### Neuron tracking using SURF and ROI overlay

**Figure 4:**
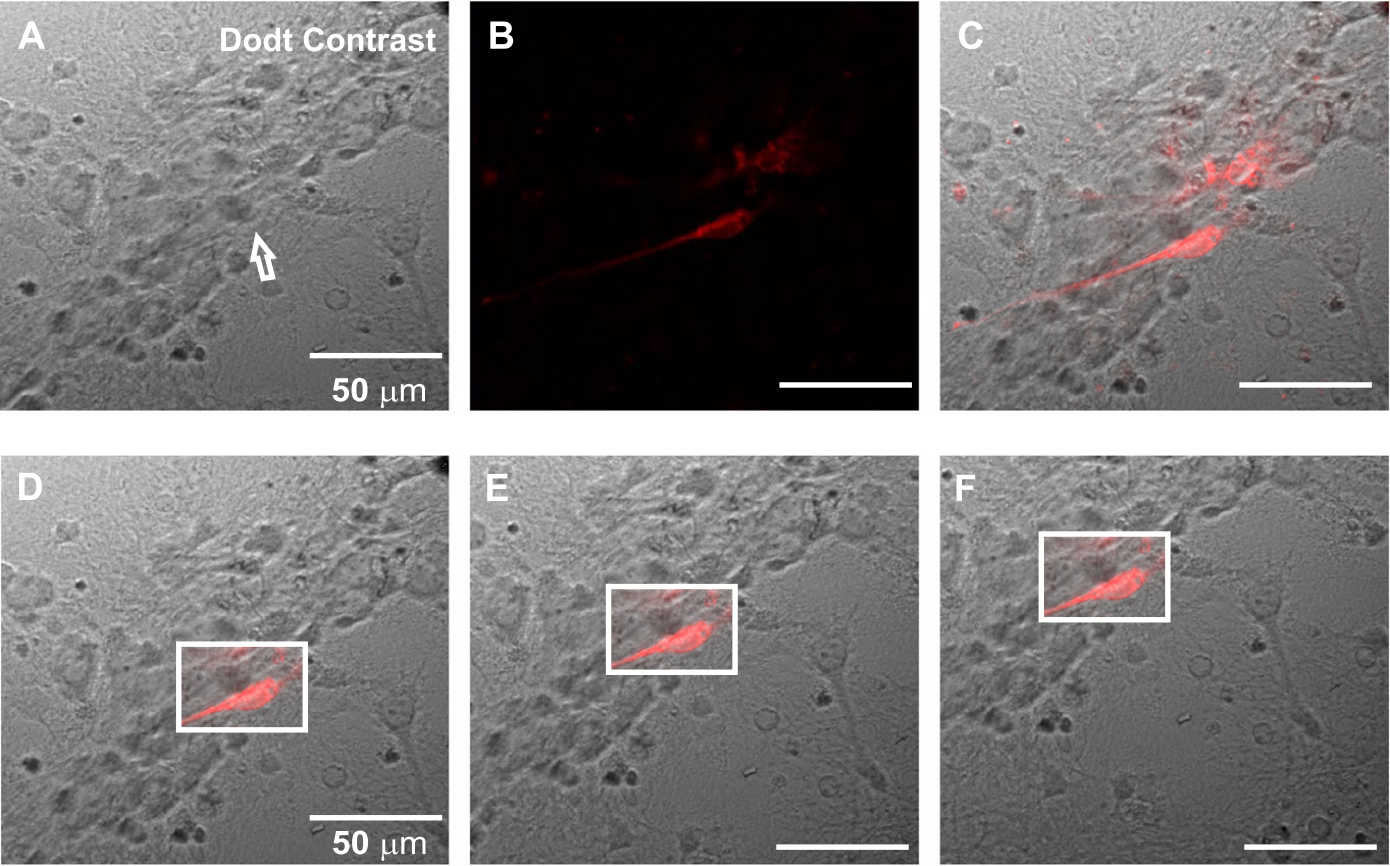
**Alpha blending and neuron tracking.(A)** Image obtained from Dodt Contrast Microscopy. The arrow indicates a neuron of interest. **(B)** Image of the cellular response to fluorescent light on the same region of **(A)**. **(C)** Real time overlay using Alpha Blending technique between **(A)** and **(B)**. The white square in **(D)**, **(E)** and **(F)** represents the neuron tracking applied to three displaced images. Blending between fluorescence information and ROI is dinamically performed and highlighted in red.

**Figure 5:**
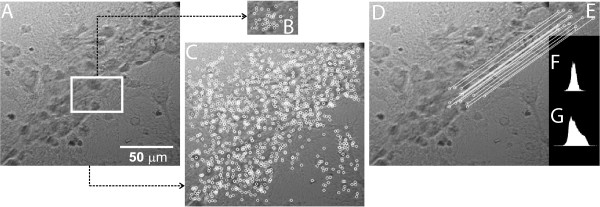
**SURF algorithm.(A)** Image with a ROI selected (white square) acquired by user. **(B)** and **(C)** SURF keypoints detected in the images. **(D)** and **(E)** Matching of the best keypoints. **(F)** and **(G)** Histograms of 8-bits grayscale images (**E** and **D**, respectively).

To maintain fluorescence information available during live preview, it is necessary to find fluorescent cells. This is accomplished by a manual search in the xyz axis in alternation with the on/off procedure at the light source for excitation in the desired region. Figure 4A and 4B show a region that is responding to light excitation appropriately. Figure 4C presents blending between fluorescence information of Figure 4B (Light on) and the image of the same region in Figure 4A (Light off). Figure 4D and 4F show the blending proccess according to a ROI (white square) tracking using SURF in displaced images.The Operation of SURF in images of cell cultures is shown in Figure 5. The ROI is selected in Figure 5A, for the subsequent detection of interest points (keypoints) at the ROI in Figure 5B and full frame in Figure 5C. In this way, we can compare the feature vector selected (normalized in white circles) in both Figure 5D and 5E, and thus select the best matches.

#### SURF performance

Some metrics were applied to the images of the built dataset to test operation under conditions similar to those found in patch-clamp experiments. Focus changes can be accomplished by applying linear filters in images. For this purpose, we used Gaussian blur technique that consists in convolving an image *f*(*x*,*y*) with a Gaussian function *h*(*k*,*l*), according to (9) [19].

(9) $$g(x,y)=\underset{k}{\sum }\underset{l}{\sum }f(x+k,y+l)h(k,l)$$

In a simplified form:

(10) g(x,y)=f(x,y)∗h

The brightness and contrast adjustments in an image *f*(*x*,*y*) can be performed from the multiplication (*α*) and addition (*β*) of constants, as shown in (11) [17].

(11) g(x,y)=αf(x,y)+β

**Figure 6:**
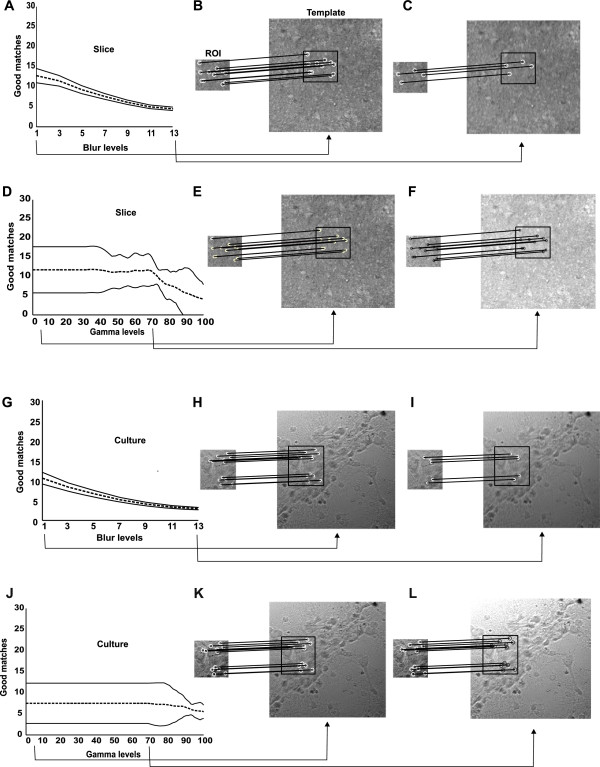
**SURF robustness in adverse image conditions.(A)** Mean amount of good matches (black line) and 95% CI (dashed lines) at different bluring. Examples of ROI detection for 1 **(B)** and 13 **(C)** levels of blurring. levels for slice images; **(D)** mean amount of good matches (black line) and 95% CI (dashed lines) at different levels of gamma (brightness). **(E)**, **(F)** Examples of ROI detection in levels 5 and 70 of brightness, respectively. **(G-L)**Same as A-F for culture images.

It is important to test SURF method in adverse conditions to validate its use in microscopy experiments, more specifically during patch-clamp. Frequently, video acquisition is noisy and suffers from changes in brightness or focus that can interfere with the tracking algorithm. To test the consistency of the SURF algorithm in such situations, we emulate changes in brightness and focus by changing gamma and blur levels of slice and culture images. For these analysis, we randomly selected 10% of our data set (Table 1). A ROI was attributed to each single image and up to 13 levels of gaussian blurring or 100 levels of gamma were applied to the image. Figure 6 shows a selected ROI and its matching with templates in normal conditions (Figure 6B and 6C), with gaussian blurring at 13 levels (Figure 6H and 6I), using gamma at 5 levels (Figure 6E and 6K), and high gamma level (factor 70) (Figure 6F and 6L). Figure 6A and 6G represent the mean amount of good matches (black line) and the 95% confidence interval (CI) (dashed lines) during blurring increase in slice and culture images, respectively, using (10). Figure 6D and 6J represent the mean amount of good matches (black line) and the 95% CI (dashed lines) during brightness increase in slice and culture images, respectively, using (11). The reliability of the SURF algorithm decayed monotonically when blur levels were increased in both slice and culture image sets (Figure 6). On the other hand, changes in SURF performance was just observed in gamma levels greater than 70 in slices while, in culture images, the performance was not affected by gamma changes (Figure 6). This data shows that ROI tracking remains uncompromised during typical changes in brightness and focus in patch-clamp experiments.

**Figure 7:**
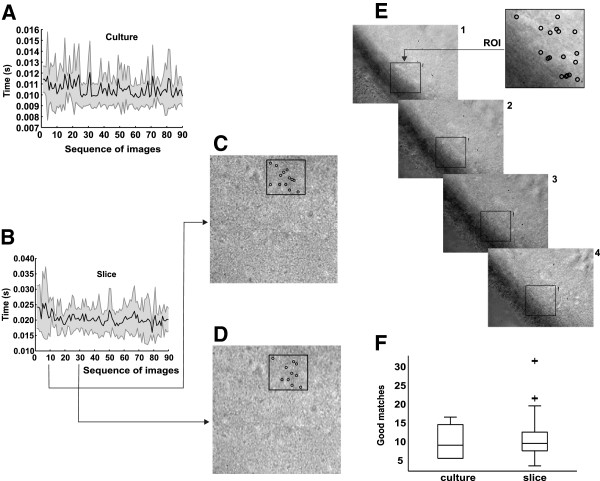
**Video-rate frame by frame detection using SURF.(A)**, **(B)** Mean time (black line) and 95% CI (gray shadow) for the detection of keypoints in culture and slice images sequences, respectively. **(C)**, **(D)** Examples of ROI detection (black square) and good matches (black circles) at different time points. **(E)** Examples of ROI detection in 4 levels of manual focus changes. **(F)** Mean amount of good matches in culture and slice image sequences.

We then applied the SURF algorithm to hippocampus culture and slice images of the entire data set (Table 1). In both cultures and slices, the ROI was rapidly retrieved within an image (Figure 7A-D). Figure 7E is a sample of ROI detection in 4 levels of manual focus changes. Figure 7F shows the boxplot graphs of medians of good matches to both types of images analysed under normal conditions. The time necessary for ROI tracking in live image is sufficient for allowing live video preview at video rate with no perceivable jerking (Figure 7A-D). While detection time was shorter in culture images compared to slices, SURF performance was similarly reliable in slice and culture videos (Figure 7F). In all tests performed, the ROI is maintained within the field of view, however, it is possible to use a minimal amount of keypoints to estimate whether the ROI is present or not.

## Discussion and conclusions

While the object tracking SURF algorithm has been widely employed in computer vision problems its usage in videomicroscopy, specially in live tissue, has been limited [20]. Here, we used the SURF method to track neurons targeted for patch-clamp in live brain slices or cultures. Once a cell is visually identified, the image acquisition program will track the cell while the microscope stage is moved in the xy axis and detect changes in focus, signaling when the cell enters the focal plane. If the neuron of interest is also labeled with fluorescent proteins (or compounds), a fluorescent image (mask) can be overlaid in real time with the live video using alpha blending. When the cell (or cells) of interest is in focus, the tracking algorithm shifts the mask in the xy axis to precisely align the acquired fluorescence image with the live video. The method reliably detected cells of interest in images acquired with adverse light conditions, high level of scatter and high background.

Acute brain slices and primary cultures were used to test the robustness of the detection algorithm in identifying reference neurons after moving the microscope stage or changing the focus. The SURF detector showed a remarkable performance when the region containing the cell of interest was moved across the sensor without slowing image acquisition. The detector also performed well in detecting changes in focus in both types of image. It is important to note here that acute slices used in patch-clamp experiments are very thick (>200 *μ*m), therefore, the image sensor register a mixture of focused cells with blurred structures from defocused planes. Hence, the detection algorithm deals with complex images containing high contrast regions blended with low-contrast ones. Changes in focus shift sharp regions as new structures enter the focal plane and blur the region (cell) of interest, creating, statistically, a whole new image. The SURF method is known to overcome affine transformations (scaling and in-plane rotation) [21], brightness and small levels of focus. Yet, this technique does not support large differences in focus. This weakness of the SURF in relation to object recognition in conventional computer vision task represent an advantage of the method applied to imaging of live thick slices as the incapacity of the SURF to detect the targeted neuron when the focus is changed indicate that that neuron is out-of-focus. Besides its computational cost is demonstrably reduced when compared with similar techniques as SIFT [22] making it very appropriate for real time image analysis.

During in vitro patch-clamp experiments microscope stage and the focus knob often moved in order to place patch pipettes. The software described here ease the retrieving of targeted neurons for patch-clamp and, in case of cells labeled with fluorescent protein (or compounds), the tracking algorithm eliminate the necessity of prolonged and/or multiple exposures to high intensity fluorescence excitation light as the fluorescence image (previously acquired) is automatically aligned with the live transmission image and overlaid in the display at the user’s discretion. Fluorescence tagging is commonly for a priori identification of cell types in the patch-clamp laboratory [23]. An uncountable number of transgenic animals displaying various fluorescent proteins in different cell types can be purchased from commercial or public organisations and these animals are extremely important tools in modern neurophysiology [23,24]. In addition, there are a variety of synthetic fluorescence compounds that can also be used for the identification of glia or specific neuron types that might have a greater affinity for particular compounds, allowing specific labeling [4]. There are also various fluorescent indicators that can be used to isolate projection neurons following a previous in vivo injection of a tracer [25]. Patch-clamp recordings of labeled cells require the constant switching between fluorescence and transmission modes [26]. If the patch-clamp setup is fitted with DIC optics, the switching between fluorescence and DIC modes normally involves removing at least two optical components and/or a filter set above the objective [23]. Mechanically removing components during patch-clamp experiments often causes vibrations that can destroy established cell/electrode seals. This problem can be more traumatic when executing multiple-cell patch-clamp recordings. The Dodt contrast method does not require optical components above the objective; however, the definition of neurons, especially neurites, is generally inferior to the one obtained with DIC optics [27]. Moving a virtual fluorescence mask according to xy-plane changes in the field of view eliminates the need to switch to fluorescence mode once a field of interest is determined.

Optogenetic activation of neurons is a technique that is becoming customary in almost every neuroscience lab [10]. In general, optogenetic constructs carry the coding for fluorescent proteins to indicate cells that are expressing the rhodopsins. The excitation wavelength often coincides with the wavelength that triggers light-activated channels (e.g. channelrhodopsin 2 tagged with yellow fluorescent protein [7,10]. Inspection of the tissue using fluorescence can, therefore, cause unwanted activation of rhodopsin-expressing neurons by the microscope light source, generating overexcitation of the tissue that leads to damage [7]. Hence, in vitro patch-clamp experiments involving optogenetics can greatly benefit from a neuron tracking software.

As mentioned above, searching of suitable cells for patch-clamp experiments involves not only a constant change in xy-plane position but also in focus and light intensity. When these changes occur, there is a frequent loss of reference by the user, requiring a new fluorescence image for determining a region of interest. Electronic focus drive, xy-plane translators, filter switching and shuttering dramatically facilitate the swapping between fluorescence and transmission and the precise return to a predetermined field of view [23]. However, the high price tag of these components is not always in the reach of every electrophysiology laboratory. Hence, the software navigation technique described here may be an alternative to the expensive electronic translators and shutters, being able to significantly simplify multiple cell patching and pipette finding. Furthermore, by dramatically reducing the exposure of neurons to high excitation light intensity we can avoid the deleterious effects of long periods of fluorescence microscopy, which leads to the production of Reactive Oxygen Species (ROS) and causes photo-oxidative damage to the cells[28-30]. By reacting with proteins, lipids, and nucleic acids [31], the increase in ROS can alter the physiology of the neurons, leading to significant cell damage. Minimal exposure of cells to irradiation can also cause the production of ROS but the capacity of the cell to eliminate these species is strongly associated the shortness of the exposure [6].

In summary, we have demonstrated a new tool for tracking neurons in electrophysiology experiments. The ability of the software to detect neurons of interest was illustrated using DIC and Dodt contrast imaging of primary neuronal cultures and acute brain slices typically used in patch-clamp experiments. Besides, technique allows for the overlaying of a fmask (fluorescence) without the need to rexposure to epifluorescence illumination.

## Abbreviations

3D: Three dimensional; ACSF: Artifcial cerebrospinal fluid; CAPES: Brazilian coordination for the improvement of higher education; DIC: Diferential interference contrast; EM-CCD: Electron multiplying charge coupled device; eYFP: Enhanced yellow fluorescent protein; GFP: Green fluorescent protein; NMS: Non maximum suppression; OpenCV: Open source Computer vision; RNA: Ribonucleic acid; ROI: Region of interest; ROS: Reactive oxygen species; SURF: Speeded-up robust features; tdTomato: Tandem dimer tomato; UML: Unified modeling language.

## Competing interests

The authors declare that the research was conducted in the absence of any commercial or financial relationships that could be construed as a potential conflict of interests.

## Authors’ contributions

HMP, HM, RNL performed the experiments; HMP and RNL designed the experiments; HMP, RMSC, AMGG and RNL wrote the paper. All authors read and approved the final manuscript.

## References

[B1] Neher E Sakmann B Steinbach J **The extracellular patch clamp: a method for resolving currents through individual open channels in biological membranes** Pflugers Archiv 1978 375 2 219 228 10.1007/BF00584247 567789

[B2] Tsien RY **The green fluorescent protein** Annu Rev Biochem 1998 67 509 544 10.1146/annurev.biochem.67.1.509 9759496

[B3] Perron A Mutoh H Launey T Knopfel T **Red-shifted voltage-sensitive fluorescent proteins** Chem Biol 2009 16 1268 1277 10.1016/j.chembiol.2009.11.014 20064437 PMC2818747

[B4] Paredes RM Etzler JC Watts LT Zheng W Lechleiter JD **Chemical calcium indicators** Methods 2008 46 3 143 151 10.1016/j.ymeth.2008.09.025 18929663 PMC2666335

[B5] Leao RN Reis A Emirandetti A Lewicka M Hermanson O Fisahn A **A voltage-sensitive dye-based assay for the identification of differentiated neurons derived from embryonic neural stem cell cultures** PLoS One 2010 5 11 e13833 10.1371/journal.pone.0013833 21079795 PMC2973948

[B6] Dixit R Cyr R **Cell damage and reactive oxygen species production induced by fluorescence microscopy: effect on mitosis and guidelines for non-invasive fluorescence microscopy** Plant J 2003 36 280 290 10.1046/j.1365-313X.2003.01868.x 14535891

[B7] Leão RN Mikulovic S Leão KE Munguba H Gezelius H Enjin A Patra K Eriksson A Loew LM Tort AB Kullander K **OLM interneurons differentially modulate CA3 and entorhinal inputs to hippocampal CA1 neurons** Nat Neurosci 2012 15 11 1524 1530 10.1038/nn.3235 23042082 PMC3483451

[B8] Murphy DB Davidson MW **Differential interference contrast (DIC) microscopy and modulation contrast microscopy** Fundamentals of Light Microscopy and Electronic Imaging, Second Edition 2001 173 197

[B9] Dodt HU Zieglgänsberger W **Visualizing unstained neurons in living brain slices by infrared DIC-videomicroscopy** Brain Res 1990 537 333 336 10.1016/0006-8993(90)90380-T 2085783

[B10] Boyden ES Zhang F Bamberg E Nagel G Deisseroth K **Millisecond-timescale, genetically targeted optical control of neural activity** Nat Neuroscii 2005 8 9 1263 1268 10.1038/nn1525 16116447

[B11] Bay H Ess A Tuytelaars T Gool LV **Speeded-Up Robust Features (SURF)** Comput Vis Image Understand 2008 110 346 359 10.1016/j.cviu.2007.09.014

[B12] Horikawa K Yamada Y Matsuda T Kobayashi K Hashimoto M **Spontaneous network activity visualized by ultrasensitive Ca(2+) indicators, yellow Cameleon-Nano** Nat Methdos 2010 7 729 732 10.1038/nmeth.1488 20693999

[B13] Hilscher M Leao KE Leao RN **Synchronization through nonreciprocal connections in a hybrid hippocampus microcircuit** Front Neural Circ 2013 7 120 10.3389/fncir.2013.00120 PMC3719444 23888129

[B14] Viola P Jones M **Rapid object detection using a boosted cascade of simple features** Proceedings of the 2001 IEEE Computer Society Conference on Computer Vision and Pattern Recognition. CVPR 2001 2001 511 518

[B15] Evans C **Notes on the OpenSURF Library** University of Bristol. Technical Report CSTR-09-001. January; 2009

[B16] Neubeck A Gool LV **Efficient non-maximum suppression**18th International Conference on Pattern Recognition (ICPR’06). IEEE 2006 2006 850 855

[B17] Szeliski R Computer Vision - Algorithms And Applications 2010 New York: Springer

[B18] Fischler MA Bolles RC **Random sample consensus: a paradigm for model fitting with applications to image analysis and automated cartography** Commun ACM 1981 24 6 383 395

[B19] Russ JC The Image Processing Handbook, 6th edition 2011 USA: CRC Press

[B20] Stanciu SG Hristu R Stanciu GA **Influence of confocal scanning laser microscopy specific acquisition parameters on the detection and matching of speeded-up robust features** Ultramicroscopy 2011 111 5 364 374 10.1016/j.ultramic.2011.01.014 21349249

[B21] Gossow D Decker P Paulus D **An evaluation of open source SURF implementations** RoboCup 2010: Robot Soccer World Cup XIV 2011 Springer Berlin Heidelberg 169 179

[B22] Lowe D **Distinctive image features from scale-invariant keypoints** Int J Comput Vis 2004 2 91 110

[B23] Borgius L Restrepo CE Leao RN Saleh N Kiehn O **A transgenic mouse line for molecular genetic analysis of excitatory glutamatergic neurons** Mol Cell Neurosci 2010 45 245 257 10.1016/j.mcn.2010.06.016 20600924

[B24] Enjin A Leao KE Mikulovic S Merre PL Tourtellotte WG Kullander K **Sensorimotor function is modulated by the serotonin receptor 1d, a novel marker for gamma motor neurons** Mol Cell Neurosci 2012 49 322 332 10.1016/j.mcn.2012.01.003 22273508 PMC3306528

[B25] Clarke JD **Using fluorescent dyes for fate mapping, lineage analysis, and axon tracing in the chick embryo** Methods Mol Biol 2008 461 351 361 10.1007/978-1-60327-483-8_25 19030810

[B26] Schiefer J Kampe K Dodt HU Zieglgansberger W Kreutzberg GW **Microglial motility in the rat facial nucleus following peripheral axotomy** J Neurocytol 1999 28 439 453 10.1023/A:1007048903862 10767097

[B27] Davie JT Kole MH Letzkus JJ Rancz EA Spruston N Stuart GJ Hausser M **Dendritic patch-clamp recording** Nat Protoc 2006 1 1235 1247 10.1038/nprot.2006.164 17406407 PMC7616975

[B28] Bartosz G **Oxidative stress in plants** Acta Physiol Plant 1997 19 47 64 10.1007/s11738-997-0022-9

[B29] Foyet CH Lelandais M Kunert KJ **Photooxidative stress in plants** Physiol Plant 1994 92 696 717 10.1111/j.1399-3054.1994.tb03042.x

[B30] Wright A Bubb WA Hawkins CL Davies MJ **Singlet oxygen-mediated protein oxidation: evidence for the formation of reactive side chain peroxides of tyrosine residues** Photochem Photobiol 2002 76 35 46 10.1562/0031-8655(2002)076<0035:SOMPOE>2.0.CO;2 12126305

[B31] Halliwell B Gutteridge JMC Free Radicals in Biology and Medicine 1989 Oxford: Claredon Press

